# Protein Gas Vesicles of *Bacillus megaterium* as Enhancers of Ultrasound-Induced Transcriptional Regulation

**DOI:** 10.1021/acsnano.4c01498

**Published:** 2024-06-19

**Authors:** Vid Jazbec, Nina Varda, Ernest Šprager, Maja Meško, Sara Vidmar, Rok Romih, Marjetka Podobnik, Andreja Kežar, Roman Jerala, Mojca Benčina

**Affiliations:** † Department of Synthetic Biology and Immunology, National Institute of Chemistry, 1000 Ljubljana, Slovenia; ‡ Institute of Cell Biology, Faculty of Medicine, University of Ljubljana, 1000 Ljubljana, Slovenia; § Department of Molecular Biology and Nanobiotechnology, National Institute of Chemistry, 1000 Ljubljana, Slovenia; ∥ CTGCT, Centre for the Technologies of Gene and Cell Therapy, Hajdrihova 19, 1000 Ljubljana, Slovenia; ⊥ University of Ljubljana, Kongresni trg 12, 1000 Ljubljana, Slovenia

**Keywords:** protein gas vesicles, gas vesicle proteins, transcription, ultrasound, sonogenetics

## Abstract

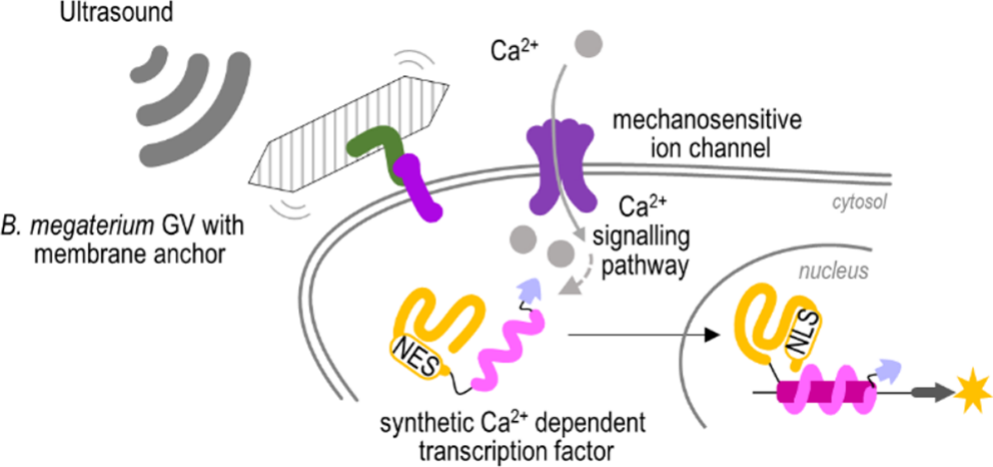

Gas vesicles (GVs) are large cylindrical gas-filled protein assemblies found in diverse aquatic bacteria that enable their adaptation of buoyancy. GVs have already been used as ultrasound contrasting agents. Here, we investigate GVs derived from *Bacillus megaterium*, aiming to minimize the number of accessory Gvps within the GV gene cluster and demonstrate the use of GVs as enhancers of acoustic radiation force administered by ultrasound. Three (*GvpR*, *GvpT*, and *GvpU*) out of 11 genes in the cluster were found to be dispensable for functional GV formation, and their omission resulted in narrower GVs. Two essential proteins GvpJ and GvpN were absent from recently determined GV structures, but GvpJ was nevertheless found to be tightly bound to the cylindrical part of GVs in this study. Additionally, the N-terminus of GvpN was observed to play an important role in the formation of mature GVs. The binding of engineered GvpC from*Anabaena flos-aquae* to HEK293 cells via integrins enhanced the acoustic force delivered by ultrasound and resulted in an increased Ca^2+^ influx into cells. Coupling with a synthetic Ca^2+^-dependent signaling pathway GVs efficiently enhanced cell stimulation by ultrasound, which expands the potentials of noninvasive sonogenetics cell stimulation.

Ultrasound has emerged as a promising noninvasive method for stimulating mammalian cells with one of its notable effects being the induction of the Ca^2+^ influx into the cytosol through various ion channels such as TRPA1, Piezo, and mPrestin.^1−3^ This Ca^2+^ influx serves as a powerful tool for controlling cellular functions, including targeted gene transcription via designed Ca^2+^-responsive transcription factors.^4,5^ Of the three primary effects of ultrasound on cells—cavitation, heating, and acoustic radiation force—the latter stands out as the least harmful effect on cell viability and can be enhanced by objects introducing acoustic contrast.^6^ Despite being effective acoustic enhancers, microbubbles^7^ face challenges related to their short retention time in the body.

An alternative strategy that is gaining momentum is the utilization of protein gas vesicles (GVs). GVs are hollow gas-filled structures with distinct acoustic properties that differ considerably from those of their liquid environment. As such, GVs have been considered acoustic reporters in bacteria and mammalian cells.^8−10^ This study focuses on recombinantly produced *Bacillus megaterium* GVs, exploring their potential as actuators of acoustic radiation force for cell stimulation. GVs are protein structures found in aquatic microorganisms^11^ that provide buoyancy to single-cell organisms to enable them to reach resources near the water surface.^12^ The GV gene clusters comprise 8 to 14 genes (*Gvp*) required for GV formation.^13^ Among them, only *B. megaterium*(14) GvpB or its homologue *Anabaena flos-aquae*(15) GvpA are found in the recently reported GV structures. Even though studies of *Halobacterium salinarum* provided insight into the binding relationships between Gvps,^16,17^ this information cannot be readily applied to GV gene clusters from other organisms to others due to the high diversity of accessory proteins. So far, only *H. salinarum*, *B. megaterium*, and *A. flos-aquae* GV gene clusters have been utilized for the recombinant production of GVs in easy-to-use bacteria like *Escherichia coli*.^8,18,19^ Among these, the GVs from *B. megaterium* were found to be the most rigid, which is owed to their small diameter.^20^

This study aims to clarify the role of proteins within the *B. megaterium* GV cluster in GV formation and evaluate the potential of GVs as amplifiers of the acoustic radiation force. The identification of redundant GV proteins led to a reduction in the number of genes required for GV production. Additionally, the study of the binding of individual Gvps to the formed GV shell provided insights into potential attachment tags at the N- and C-termini of Gvps.

Furthermore, the ability of GVs to enhance the mechanosensitivity of mammalian cells to ultrasound is investigated in this work. Since the identified *B. megaterium* Gvps did not bind with all GVs, GvpC from *A. flos-aquae* was used to bind GVs to mammalian cells. Isolated GVs were then employed as acoustic enhancers for stimulating mammalian cells with ultrasound, resulting in increased efficiency of acoustic radiation force and enhanced ultrasound signaling transduction through mechanosensitive calcium channels.

In addition, GVs anchored to the cell surface via integrins were found to effectively enhance the expression of target genes under ultrasound stimulation. These results emphasize the significant potential of GVs as effective acoustic enhancers for ultrasound-based cell stimulation and their use in noninvasive cell manipulation.

## Results

### Gene Cluster Minimization

**Figure 1:**
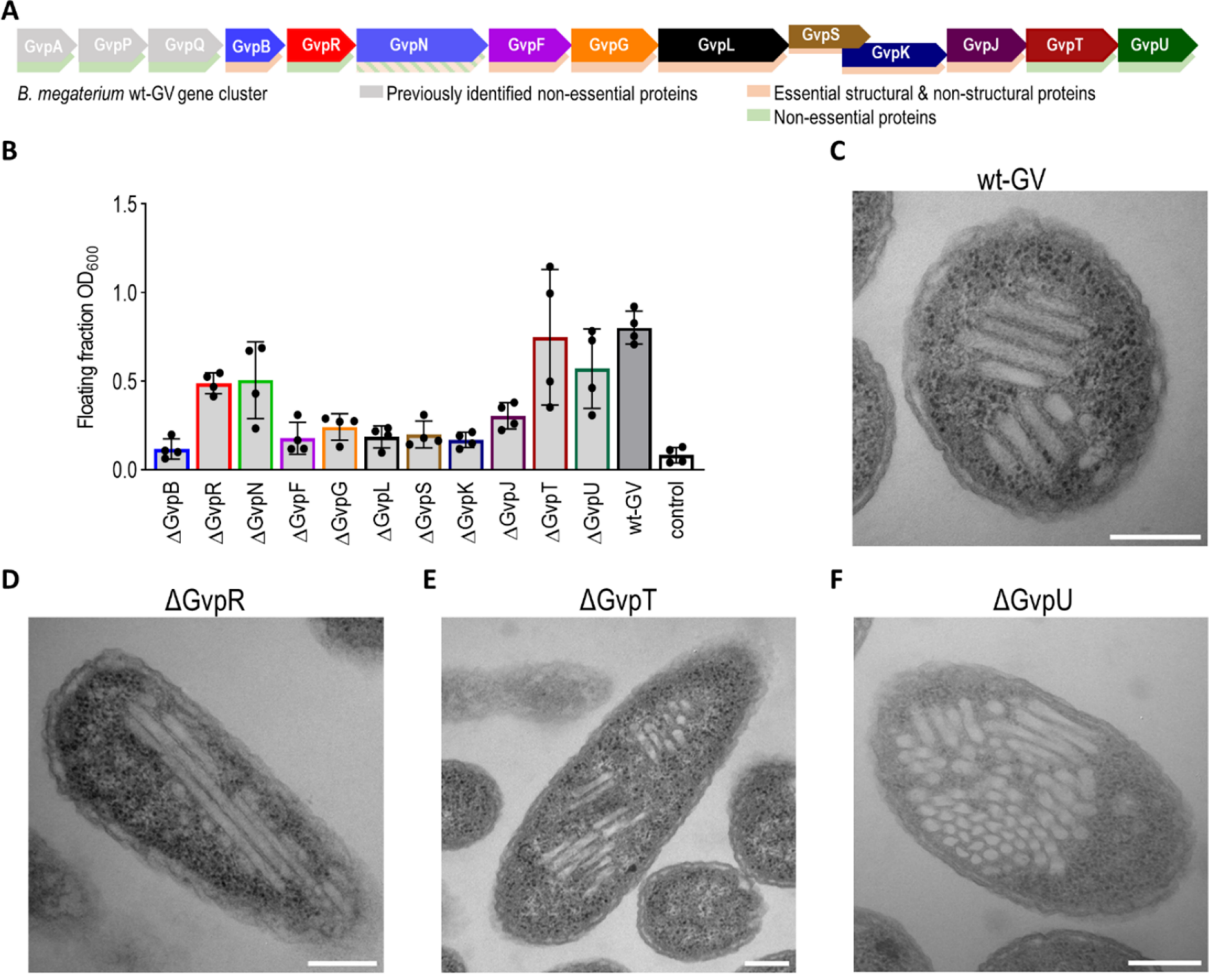
Essential proteins for GV formation from *B. megaterium* GV cluster. (A) Scheme of *B. megaterium* GV gene cluster. Genes absent in plasmid pST39-pNL29 are shown in gray. GvpN is not essential for the formation of GV, but it is necessary for their elongation. (B) Flotation assay results. Bar colors correspond to the scheme in (A). Bar represents the mean of four independent experiments indicated by dots; see also Figure S1. (C–F) Electron microscopy images of bacteria producing GVs from (C) whole cluster (wt-GV), (D) cluster without GvpR (GV^Δ*G*vpR^), (E) cluster without GvpT (GV^Δ*G*vpT^), and (F) cluster without GvpU (GV^Δ*G*vpU^). The white scale bar represents 100 nm.

The original GV gene cluster of *B. megaterium* containing proteins GvpAPQBRNFGLSKJTU was previously shortened.^19^ The plasmid pST39-pNL29, crucial for recombinant production of GV in *E. coli*, comprises 11 genes encoding GvpBRNFGLSKJTU (wt-GV). To identify the essential genes for GV formation and further reduce the cluster size, we systematically inserted stop codons into each gene, analyzing their impact on GV formation through a flotation assay (Figures 1A,B and S1). In the initial screening, *GvpR, GvpN, GvpT*, and *GvpU* were identified as possible nonessential genes. Electron microscopy confirmed GV formation in Δ*G*vpR (Figure 1D), Δ*G*vpT (Figure 1E), and Δ*G*vpU (Figure 1F), while Δ*G*vpN showed no GVs (Figure S2).

**Figure 2:**
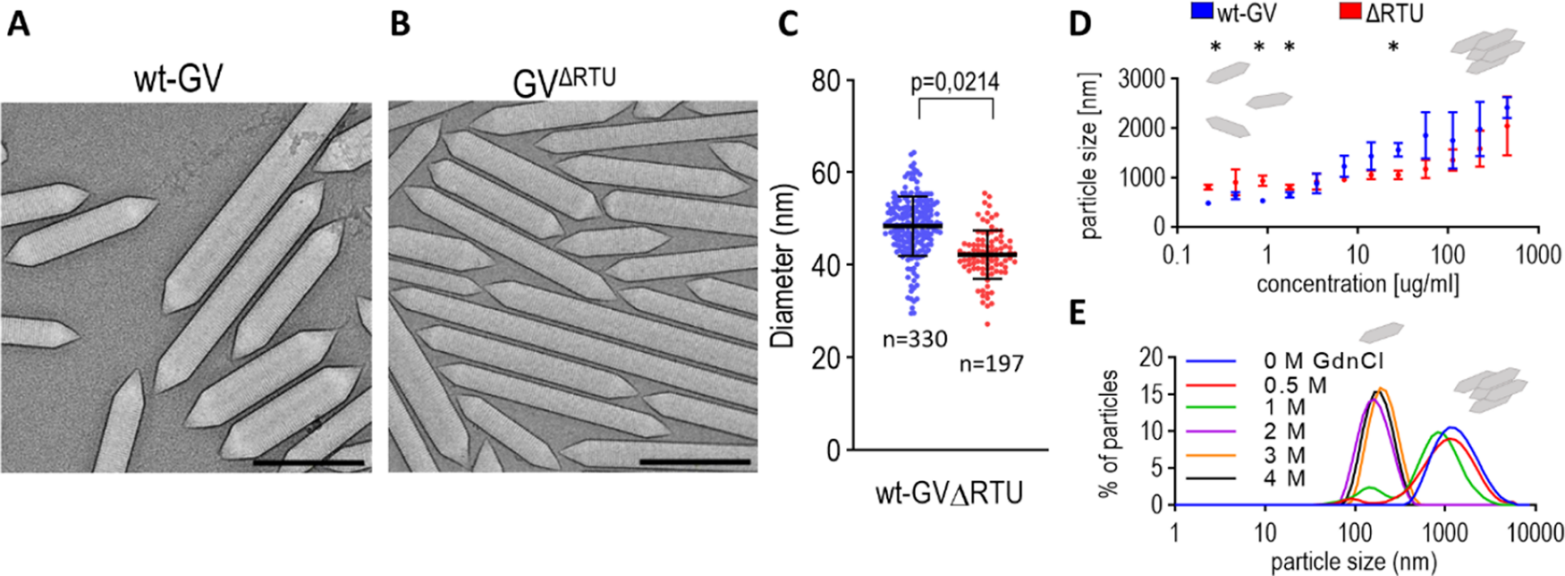
ΔRTU GVs are narrower than those of wt-GVs. The GVs isolated from *E. coli* transformed with the plasmid carrying wt-GV operon (A) or a plasmid ΔRTU (B) were imaged with cryo-EM. The black bar represents 200 nm. (C) Diameters of GVs (*n* = number of measured vesicles combined from three cryo-EM experiments). Statistical analyses and the corresponding p-values are listed in Table S4. (D) Effect of GV concentration on the clustering of wt-GVs and ΔRTU GVs. GVs were diluted with PBS. The statistical significance of the diameter difference is indicated above the graph. (E) Effect of guanidine hydrochloride on the size of wt-GV clusters. The concentration of GVs was 50 μg/mL. The curves in (D, E) show the average values of three measurements using DLS.

Next, we prepared a ΔRTU gene cluster omitting nonessential *GvpR*, *GvpT*, and *GvpU*. The resulting ΔRTU GVs were isolated from *E. coli* and imaged using cryo-electron microscopy (cryo-EM) (Figure 2A,B). The dimensions of GVs vary significantly among organisms from 100 to a few 1000 nm in length and 28 to 110 nm in diameter.^14^ The resulting ΔRTU exhibited a significantly smaller diameter (average 42 ± 7 nm) compared to wt-GVs (average 48 ± 8 nm) (Figure 2C). The length of the GVs exhibited a high degree of variability from 40 nm up to 2 μm (Figure S2).

The dynamic light scattering (DLS) revealed a tendency for *B. megaterium* GVs to form large clusters. These clusters posed a challenge for diameter measurements, prompting us to explore methods to disrupt their formation. Dilution disrupted cluster formation (Figures 2D and S3A). The comparison of the size distributions of ΔRTU reveals similar results, with variation only at concentrations below 2 μg/mL. Observing individual GVs by diluting samples is, however, not feasible for cryo-EM. To address this issue, we used a high concentration of guanidinium chloride (GdnCl), which acts as a chaotropic agent that disrupts the formation of intermolecular bonds (Figure 2E). The GV structure was not affected by GdnCl below a 4 M concentration. A weaker effect was also observed with 1 or 2 M NaCl, allowing the formation of two populations of GVs (Figure S4B). Taken together, GdnCl or NaCl most likely impacted the cluster formation by reducing electrostatic interactions between the external surfaces of the GVs. We also measured ζ-potentials of −11 ± 0.8 mV for wt-GV and −13 ± 1.5 mV for ΔRTU.

### Role of Accessory Proteins in the GV Formation

**Figure 3:**
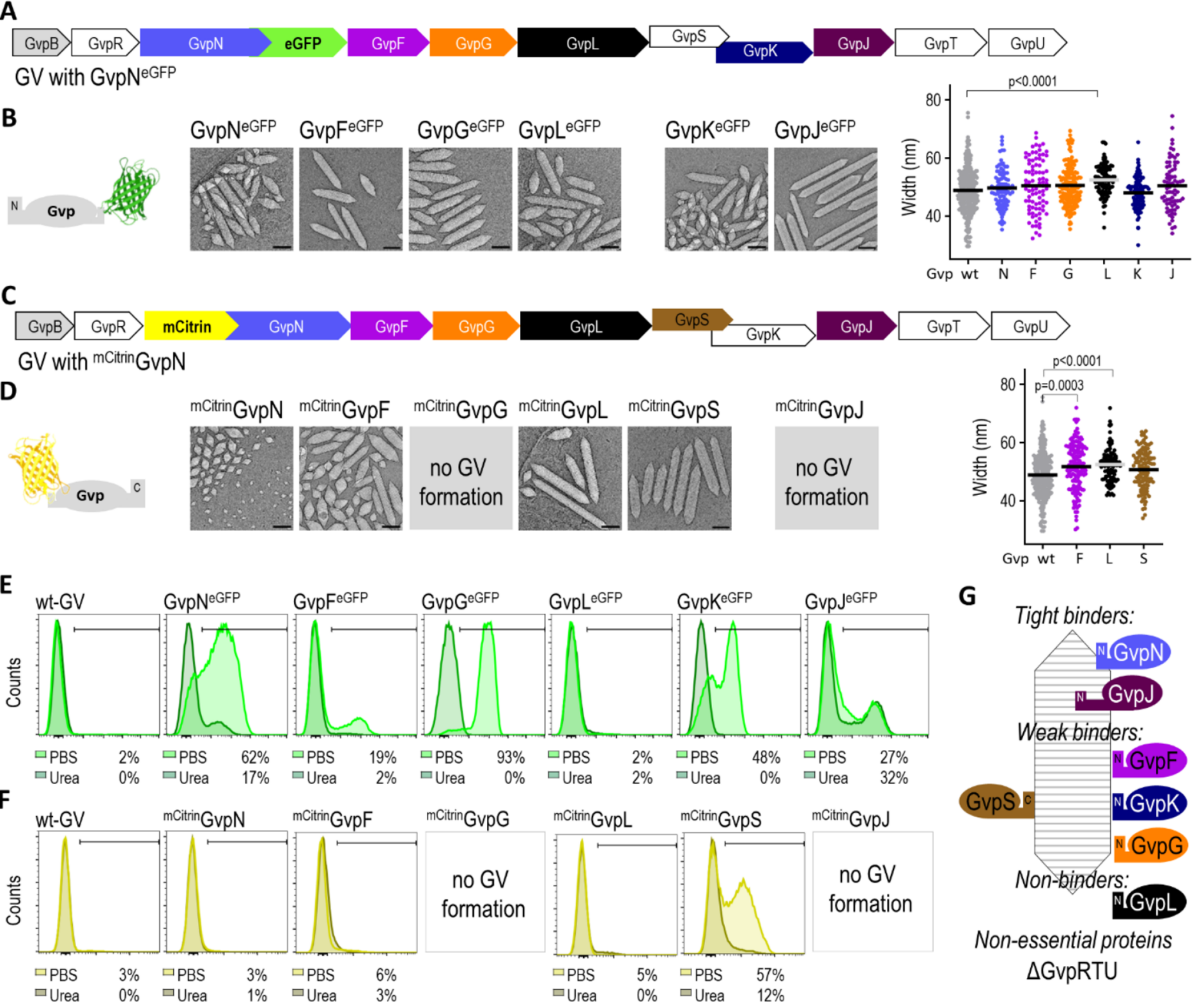
Flow cytometry of GVs produced from GV gene cluster carrying individual fluorescently tagged proteins. (A, C) Scheme of GV gene cluster with eGFP added to the C-terminal of GvpN (A) or mCitrine added to the N-terminal of GvpN (C). Note: The same approach was used for the other GV proteins indicated by color. (B, D) Cryo-EM of isolated GVs with individual fluorescently tagged Gvp protein (indicated above image). The black scale bar represents 100 nm. The diameters of cylindrical parts of GVs with tagged Gvp were determined from cryo-EM images. Statistical analyses and the corresponding p-values are given in Table S3. Note: ^mCitrine^GvpN diameters were not determined due to the absence of a cylindrical part. (E, F) Flow cytometry of isolated GVs in PBS or 6 M urea. Gates depicting the percentage of the population with fluorescent proteins are shown on the plots. The gating strategy is shown in Figures S5 and S6. (G) Scheme of the proposed interaction of Gvp with the GV shell protein GvpB.

The *B. megaterium* GV gene cluster comprises the main structural protein GvpB (also named GvpA2),^14^ nonessential GvpRTU. GvpAPQ^19^ proteins and GvpNFGLSKJ proteins (Figure 1A). Next, we investigated whether any of the GvpNFGLSKJ proteins directly interact with the GV shell structure composed of GvpB. Each of the GvpNFGLSKJ proteins from the wt-GV gene cluster was individually tagged with either a C-terminal eGFP or N-terminal mCitrin (Figure 3A,C), except for GvpK and GvpS, which exhibit a 47-bp sequence overlap. The GvpK was only tagged with eGFP at the C-terminus (GvpK^eGFP^) and GvpS was tagged with mCitrin at the N-terminus (^mCitrin^GvpS). We successfully isolated all GVs with Gvp proteins tagged at the C-terminus (Figure 3A,B) and GVs from GV gene clusters expressing mCitrin-tagged GvpNFL or S from *E. coli* (Figure 3C,D). We were unable to isolate GVs with ^mCitrin^GvpG and ^mCitrin^GvpJ. Expression of tagged Gvp proteins was confirmed by the WB (Figure S7).

The positioning of the fluorescent protein tag to Gvps differentially affected the shape of the GVs (Figure 3B,D). GVs produced from a ^mCitrin^GvpN gene cluster were small, spindle-shaped, not yet fully formed vesicles. The larger diameter than wt-GV is characteristic for GVs with ^mCitrin^GvpF, ^mCitrin^GvpL, and GvpL^eGFP^.

To test the stability of interactions between the GV shell and individual tagged Gvps, isolated GVs with tagged proteins were incubated in 6 M urea for 24 h to disrupt weak interactions. Incubation in urea has no effect on eGFP and mCitrin fluorescence^21^ and structural characteristics of GVs.^22^ Flow cytometry confirmed the fluorescence profiles of GFP and mCitirine and indicated the interactions between GVs and Gvps (Figures 3E,F and S5, gating strategy). The GvpL^eGFP^, ^mCitrin^GvpN, ^mCitrin^GvpF, and ^mCitrin^GvpL did not bind to GVs. The GvpF^eGFP^, GvpG^eGFP^, GvpK^eGFP^, and ^mCitrin^GvpS bound to GVs but were released from GV after incubation with urea. The GvpJ^eGFP^ and to some extent GvpN^eGFP^ retained fluorescence after incubation with urea.

Isolation of GVs showed that none of the C-terminally tagged Gvps and N-terminally tagged GvpNFLS hindered the functional formation of GVs (Figure 3G). Observations by flow cytometry showed that only C-terminally tagged GvpNFGKJ and N-terminally tagged GvpS were detected as GV-bound. GvpL did not bind to the GVs. The binding of GvpN, GvpF, and GvpG depends on their N-termini. Labeling at the N-terminus of GvpG affected GV synthesis indicating the importance of the protein in GV synthesis. Although GvpL, S, and K are essential, these proteins were not incorporated into the GV structure. The GvpJ was tightly bound or incorporated into the GV structure with the N-terminus (Figure 3G).

To further enhance the understanding of the role of GvpJ we tagged GvpJ^eGFP^ with an additional C-terminal tag apoferritin which allows visualization with Cryo-EM^23^ (Figure S8). The apoferritin structures were exclusively found in the cylindrical part of the GVs around the polarity inversal point. From GV structures,^14,15^ it is elucidated that the GvpJ is not a structural protein of the GV vesicle. Based on the location of GvpJ around the polarity inversal point, it indicates its involvement in elongation likely adding GvpB units into the growing GV.

### Functionalization and Targeting of GVs to Mammalian Cells

Our initial attempt to utilize accessory Gvps from the *B. megaterium* gene cluster for attachment to mammalian cells faced limitations due to weak or partial binding of Gvps to GV. We therefore extended our Gvp protein array to gene clusters from other organisms. GV-producing organisms often coexpress a structural protein known as GvpC, which adheres to the outer surface of gas vesicles, reinforcing their structure and rendering them 3 times more resistant to hydrostatic pressure in the case of *A. flos-aquae*.^24^ GvpC also serves as a platform for GV functionalization by allowing the attachment of various tags to its N- and C-terminus, facilitating the binding of GVs to cells.^14,25^

**Figure 4:**
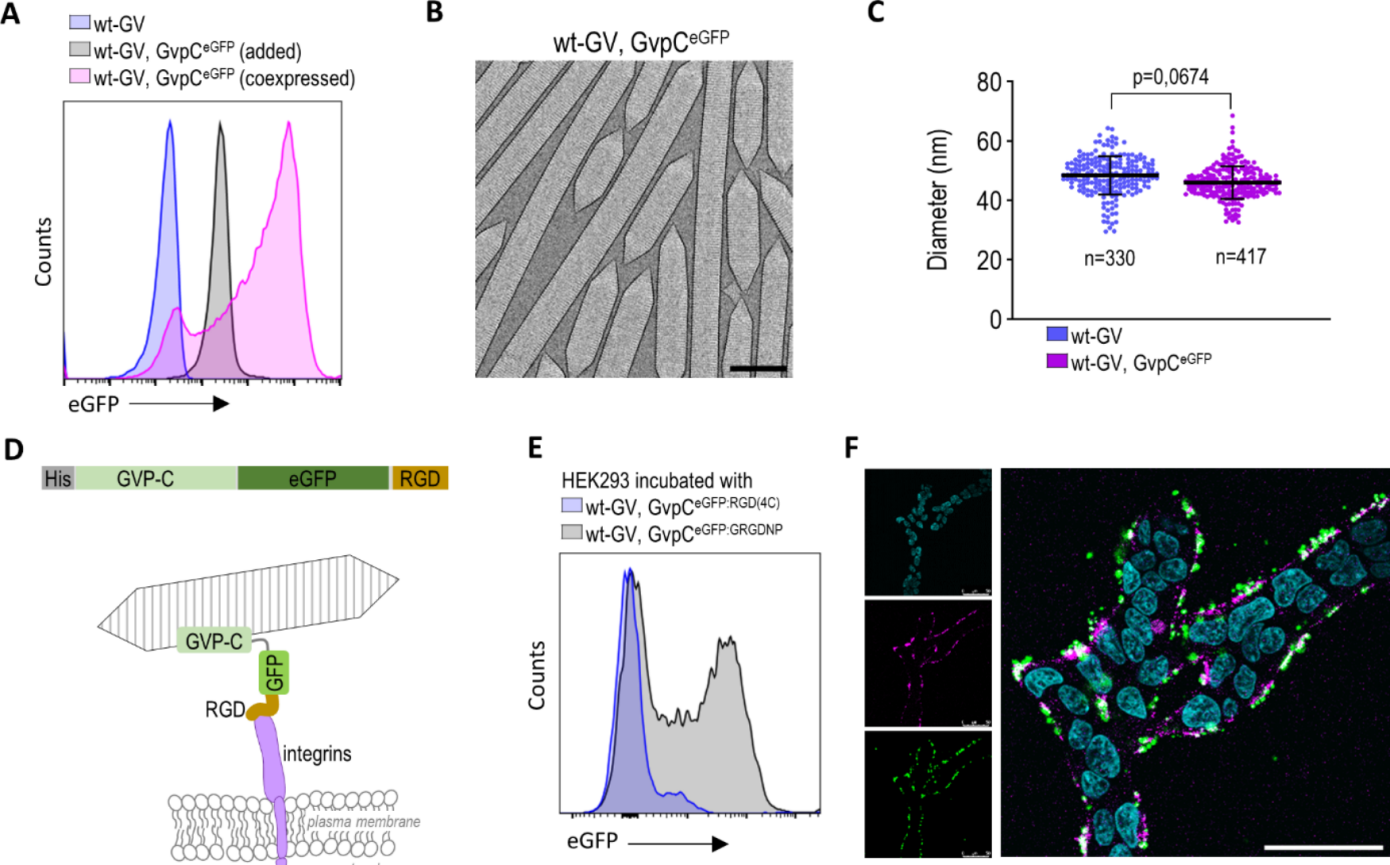
Anchoring GVs to the cell surface integrins is mediated by GvpC tagged with integrin-binding sequences. (A) Flow cytometry of wt-GVs, wt-GV with coexpressed GvpC^eGFP^ or wt-GVs with added GvpC^eGFP^. The gating strategy is shown in Figure S9. (B) Cryo-EM of wt-GVs with coexpressed GvpC^eGFP^. Bar represents 100 nm. (C) Comparison of wt-GV and wt-GV, GvpC^eGFP^ diameter. Cryo-EM images were used for the measurements. *n* = number of measured GVs from three independent cryo-EM experiments. Statistical analyses and the corresponding *p*-values are listed in Table S3. (D) Scheme of the GvpC construct, which is linked to the eGFP and an integrin-binding site RGD, and binding of GVs with GvpC^eGFP:RGD^ to integrins on the cell surface. (E) Flow cytometry of HEK293 cells incubated with GVs with GvpC^eGFP:RGD(4C)^ or GvpC^eGFP:GRGDNP^. The gating strategy is shown in Figure S9. (F) Image of HEK293 cells incubated with GVs with GvpC^eGFP:GRGDNP^. Legend: Hoechst dye (blue) nuclei; Alexa 633 B-subunit of cholera toxin (purple), membrane, and GVs with GvpC^eGFP:GRGDNP^ (green). The scale bar is 50 μm.

To enable the functionalization of *B. megaterium* GVs on mammalian cells, we investigated the binding capability of GvpC from *A. flos-aquae* to *B. megaterium* GVs. The C-terminally eGFP coupled GvpC (GvpC^eGFP^) was synthesized, isolated, and added to isolated wt-GVs or coexpressed simultaneously with wt-GVs. Flow cytometry analysis revealed improved GvpC binding to wt-GV with coexpression (Figure 4A). GVs with attached GvpC retained the shape and diameter of wt-GVs (Figure 4B,C).

For anchoring GVs to mammalian cells, GvpC was tagged with an integrin-binding peptide (Figure 4D). Two RGD peptide sequences, ACDCRGDCFC (or RGD(4C)) and GRGDNP were selected. The RGD(4C) peptide was previously shown to bind GVs to mammalian cells.^25^ RGD(4C) (GvpC^eGFP:RGD(4C)^) had minimal binding to HEK293 cell surface, possibly due the predominant expression of α5β1 integrins, which have a different binding site than αvβ3 integrins interacting with the RGD(4C) sequence which are present on U87 cells.^26,27^ The GVs with GRGDNP (GvpC^eGFP:GRGDNP^) effectively bound to cells, confirming the specificity of the GRGDNP sequence for integrins expressed at the HEK293 cell surface (Figure 4E,F).

### GVs as Acoustic Force Enhancers

**Figure 5:**
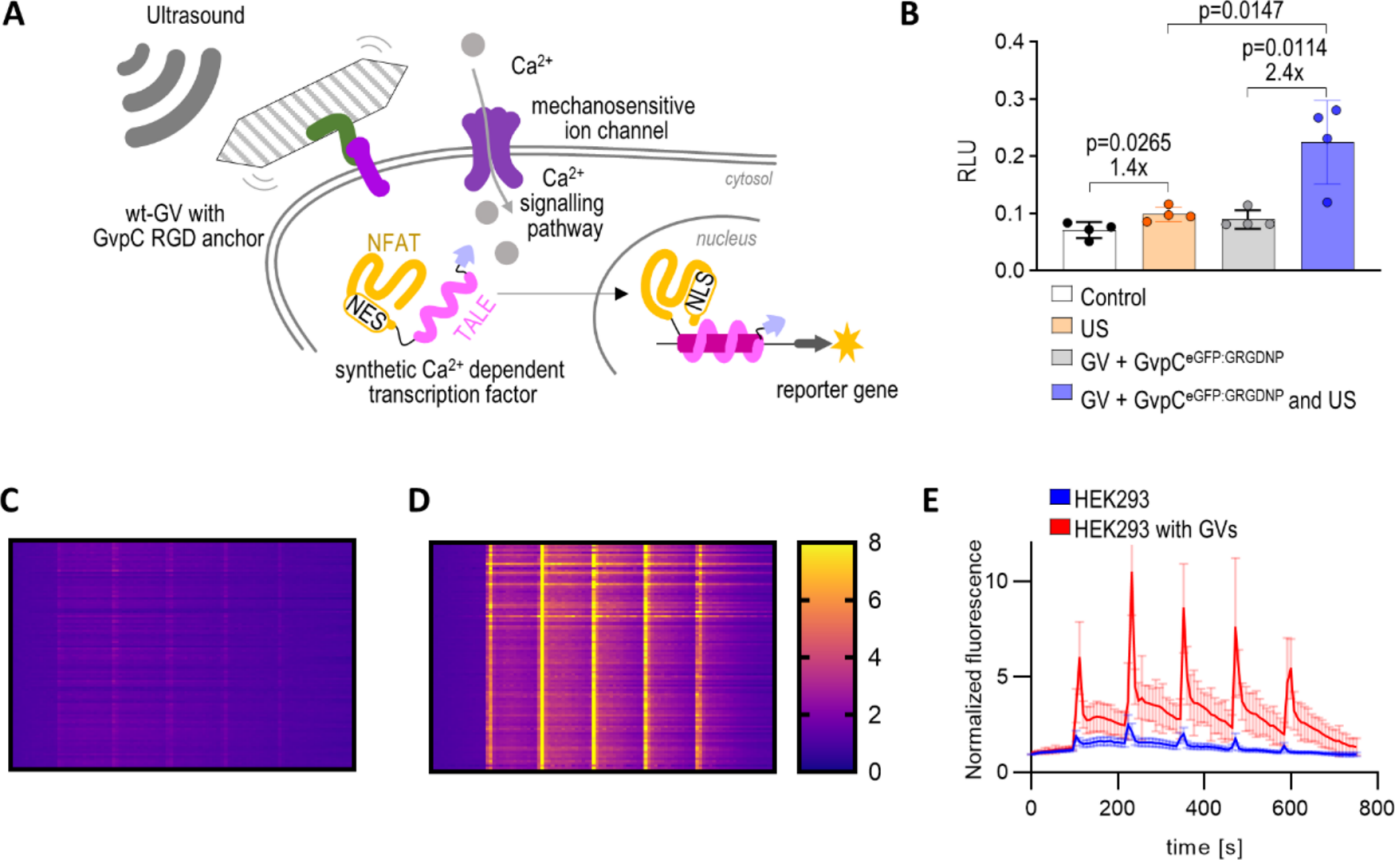
Membrane-anchored GVs actuate acoustic pressure. (A) Scheme of signaling ultrasound stimulation of mammalian cells. Ca^2+^ influx causes dephosphorylation of designed calcium-dependent NFAT-based transcription factors, which translocates into the nucleus and induces transcription of the target gene. (B) Ultrasound stimulation (US) of HEK293 cells incubated with GVs with GvpC^eGFP:GRGDNP^. In samples with GVs, the culture medium was removed prior to stimulation and GVs were added for 15 min, after which 4 mL of culture medium was added. Ultrasound was applied for 2 h and the expression of luciferase reporter was analyzed 4 h after stimulation. The bars represent the mean ± s.d.; *n* = 4 biologically independent cell experiments. Statistical analyses and the corresponding *p*-values are listed in Table S3. The amounts of transfected plasmids are listed in Table S2. Ca^2+^ influx measurements were performed with HEK293 cells without GVs attached (C) and cells with GVs attached (D). Heat maps represent normalized Fura2-TH fluorescence of 100 ROI in the field of view (Figure S10). Data of mean values ± s.d. are compared in (E).

Due to their hollow nature, GVs exhibit distinct acoustic properties, which affect their response to acoustic stimulation.^28^ Since acoustic stimulation has the ability to open mechanosensitive channels and induce Ca^2+^ current into the cytosol without acoustic enhancers,^1−3^ we speculated that the addition of GVs to the membrane would enhance this effect (Figure 5A). To explore this, HEK293 cells were transfected with genetically engineered calcium transcription factors based on NFAT (CaTF)^4^ along with the firefly luciferase reporter. The transfected cells were incubated with integrin-binding GVs. We stimulated the cells with a 1 MHz pulsed ultrasound for 2 h. The results demonstrated significant enhancement in luciferase expression compared to cells without GVs (Figure 5B).

To validate that the observed effect was attributed to an increased Ca^2+^ influx, we monitored HEK293 cells using Ca^2+^-sensitive dye Fura2-TH.^29^ The normalized signal of the GV-bound cells clearly indicated a more pronounced elevation in the Ca^2+^ concentration upon ultrasound stimulation (Figure 5C–E). GVs anchored to mammalian cells through modified GvpC as a mediator can thus act as an acoustic force actuator, enhancing the Ca^2+^ influx, which can be coupled to the translation of in principle any selected protein.

## Discussion

The investigation into the *B. megaterium* gene cluster revealed redundant Gvp proteins, leading to a reduction of the essential genes required for GV production. In addition to the previously identified redundant proteins GvpAPQ^19^ and GvpRT,^9^ we also identified GvpU as dispensable for the formation of functional *B. megaterium* GVs produced in *E. coli*, confirming a recent discovery in this field.^30^ Removal of the three nonessential Gvps reduces the cluster to the same size as that of eight essential genes from the GV cluster in *Haloferax vulcanii*,^31^ emphasizing the modular nature of GV gene clusters across organisms. A comparison of the clusters shows that in addition to the main structural protein (GvpA or GvpB), the Gvps FGLKJ are found in both clusters. Accessory GvpM from *H. vulcanii* and GvpS from *B. megaterium* show similarities to GvpA.^13^ GvpO and GvpN seem to be the only unrelated proteins in these clusters. Deletion of GvpR, GvpT, and GvpU, although nonessential, reduced GV diameter by approximately 15%, making the already narrowest vesicles even narrower.^20^ While such structures have a high resistance to collapse under hydrostatic pressure, this property also hinders their use in ultrasound imaging as they do not buckle under acoustic forces^24^ but might be useful as more stable scaffolds. The higher stability also resulted in longer GVs being found in the RTU samples. The wt-GVs of *B. megaterium* tend to form concentration-dependent aggregates that can be disrupted by the chaotropic agent guanidinium chloride or NaCl. ζ-Potential measurements confirm the tendency of wt-GVs for aggregation.^32^ Deletion of the three nonessential genes gives comparable results, but the individual GVs appear to have a larger hydrodynamic radius. This could be due to possibly longer GVs that remain stable because of a narrower and thus a more rigid shell. Despite previous reports, we could not confirm the effect of GvpU on clustering.^30^ These results highlight that *B. megaterium* GVs behave differently from those of *A. flos-aquae*, which cluster at salt concentrations above 1 M.^33^

Understanding individual interactions of Gvp with the GV shell is pivotal for functionalization. Blocking the N-termini of GvpG and GvpJ prevented GV formation, demonstrating their essential role. Weak GvpNFGK and GvpS binding to the GV shell and strong binding of GvpJ were elucidated, although GvpJ has not been localized in the recently determined GV structure. Cryo-EM images localized the ferritin-bound GvpJ to the cylindrical parts of GVs. We noticed their proximity to polarity inversial point, which could indicate that GvpJ plays an important role in the addition of new GvpB units to the growing GV. GvpJ may also have the equivalent function as GvpM in *H. vulcanii*, as the proteins share some sequence similarities.^16^ GvpN N-terminus tagging resulted in the formation of spindle-shaped GVs, lacking an elongated cylindrical part. This could be explained by the role of GvpN in the formation of GVs. Previous studies have shown that the deletion of GvpN in *Serratia* sp. also results in the production of spindle-shaped bicones.^34^ As an ATPase,^35^ GvpN could be the determining factor for the formation of the cylindrical part of the GVs.

Hollow structures are differently affected by ultrasound stimulation than the cells due to their acoustic properties. Since ultrasound penetrates deeper in tissue than light, it provides a promising method for remote control of cell therapies. In comparison to microbubbles that have been used as acoustic contrasting agents,^36^ GVs retain their structural integrity far longer.^7,37^ Because *B. megaterium* GVs are small in diameter compared to GVs from other organisms, they do not buckle or collapse by 1 MPa ultrasound. As such, they are subjected to an acoustic radiation force, which is translated into a mechanical force exerted on the membrane due to GVs binding to it. An integrin-binding RGD motif tethered to GvpC was used to anchor GVs to mammalian cells. The RGD or R8 peptides tethered to GvpC enable binding to mammalian cells.^25^ We confirmed that the *A. flos-aquae* GvpC binds to the *B. megaterium* GvpB shell, providing GVs anchoring to mammalian cells. This also shows that Gvps are somewhat interchangeable, as nonstructural proteins of *B. megaterium* have also been shown to enable the formation of GVs made of *A. flos-aquae* GvpAs.^38^

Anchored to cell surfaces via integrins, GVs enhanced the acoustic radiation force efficiency, presenting them as prospective mediators for ultrasound-induced cellular regulation. The binding of GVs to target integrins allows us to transfer the mechanical forces of the ultrasound to the cytoskeleton.^39^ These in turn open mechanosensitive Ca^2+^ channels and induce a strong Ca^2+^ current. The Ca^2+^ influx, which is associated with calcium-responsive NFAT-based transcription factors,^4^ can be used to regulate target genes. Ultrasound stimulation clearly shows that the addition of GVs to transfected cells improves ultrasound-induced transcriptional activation from 1.4- to 2.4-fold activation, highlighting their potential in sonogenetics. The effect was also visible using Ca^2+^-responsive dyes. Ultrasound parameters should be further investigated, as different amplitudes and frequencies may have an even greater effect.

This work advances our understanding of accessory proteins in GV formation and provides insight into the functionalization and applications of *B. megaterium* GVs in sonogenetics. The molecular insights into Gvp proteins and GVs not only contribute to our fundamental understanding of these biological structures but also offer promising avenues for advancements in biotechnology, nanomedicine, and cellular engineering.

## Materials and Methods

### Cloning and Plasmid Construction

Plasmid pST39-pNL29 was a gift from Mikhail Shapiro (Addgene plasmid # 91696; http://n2t.net/addgene:91696; RRID: Addgene91696). All plasmids were constructed using the Gibson assembly method.^40^

### Gene Cluster Minimization and Flotation Assay

A STOP codon was introduced into each of the genes of the pST39-pNL29 GV gene cluster using the oligonucleotides listed in Table S1. *E. coli* Rosetta RARE were transformed using knocked-out plasmids and grown overnight in 10 mL of LB broth supplemented with ampicillin and 0.8% v/v glucose. Bacteria were then seeded in LB broth supplemented with ampicillin and 0.08% v/v glucose to OD_600_ around 0.1 and grown at 37 °C. At OD_600_, 0.6 mM IPTG was added, and bacteria were incubated overnight at 30 °C. After that, 5 mL of the bacteria were put into high test tubes with 1 cm diameter, and 5 mL of 150 mM NaCl was added. After 24 h, 1 mL below the surface was taken and OD_600_ was measured.

### GV Production

Gas vesicles were produced in *E. coli* Rosetta RARE using a modified preparation protocol.^41^ GVs were purified with 3 steps of 1 h centrifugation at 350*g* at 4 °C, each time transferring the floating phase into PBS. GV concentration was determined with absorbance measurement at 500 nm in accordance with the preparation protocol.^41^

### GvpC Isolation

Protein his-GvpC^eGFP^ was produced in *E. coli* BL21 (DE3). Protein was extracted from the cell lysate using Ni-NTA affinity chromatography (high-density nickel and agarose bead technologies). Purification was performed using size exclusion chromatography (Superdex 75, Cytiva). Isolated his-GvpC^eGFP^ was kept in PBS buffer at 6 °C.

### GvpC Binding to *B. megaterium* GVs

We prepared purified GVs to a concentration of OD_500_ = 1 and added isolated GvpC^eGFP^ to the molar ratio 1:5 in favor of GvpC^eGFP^. We incubated the solution for 1 h at room temperature and then purified GVs with one cycle of centrifugation for 1 h at 350*g* and 4 °C.

### Cell Culture

The embryonic kidney HEK293 cell line (ATCC) was cultured in Dulbecco’s modified Eagle’s medium (DMEM; Invitrogen) supplemented with 10% fetal bovine serum (Gibco) at 37 °C in a 5% CO_2_ environment.

### Transfection

For confocal microscopy experiments, 5 × 10^4^ HEK293 cells were seeded per well in an eight-well chamber slide (Ibidi). For ultrasound stimulation experiments, 4 × 10^5^ HEK293 cells were seeded in 35 mm glass bottom Petri dishes (Cellvis). At 50–70% confluence, HEK293 cells were transfected with a mixture of DNA and polyethylenimine (PEI, linear, MW 25 000; Polysciences, catalog no. 23966). Per 500 ng DNA, 6 μL of PEI stock solution (0.324 mg/mL, pH 7.5) was used. Amounts of transfected plasmids are listed in Table S5. An empty pcDNA3 plasmid (Invitrogen) was used to equalize the total DNA amounts under different experimental conditions.

### Flow Cytometry

Isolated fluorescently labeled GVs were analyzed with Aurora spectral cytometer with three lasers (Cytek). For experiments with urea, the GV solutions were split, and half was incubated in 6 M urea in PBS for 24 h before flow cytometry analysis. HEK293 cells incubated with fluorescently tagged GVs were first solubilized by pipetting in PBS. GVs were added to the cells at the final concentration of OD_500_ = 2. The mixture was incubated for 30 min at 37 °C prior to flow cytometry analysis.

Results were analyzed using FlowJo (TreeStar Ashland, OR) and SpectroFlo (Cytek).

### Immunoblotting

GV proteins of isolated GVs were separated on 10% SDS-PAGE gels (200 V, 45 min) and transferred to a nitrocellulose membrane (350 mA, 60 min). Membrane blocking, antibody binding, and membrane washing were performed using an iBind Flex Western device (Thermo Fisher) according to the manufacturer’s protocol. The primary antibodies were rabbit-anti-GFP (Invitrogen A11122; diluted 1:1000). The secondary antibodies were HRP-conjugated goat antirabbit IgG, diluted 1:2000 (Abcam ab6721). The secondary antibodies were detected with an ECL Western blotting detection reagent (Super Signal West Femto; Thermo Fisher) according to the manufacturer’s protocol.

### DLS and ζ-Potential

For DLS experiments, we used the concentration of GVs in PBS at OD_500_ = 2.05 (300 mg/mL) if not stated otherwise. The reagents used were PBS, 6 M GdnCl in PBS, or 4 M NaCl in MQ. During dilution experiments, the concentration was confirmed using OD_500_. For ζ-potential measurements, we used the concentration of GVs in PBS at OD_500_ = 1.2 (175 mg/mL). ζ-Potential results are the average of 6 measurements. DLS and ζ-potential measurements were performed using Zetasizer Nano (Malvern).

### Confocal Microscopy

For the analyses of GVs binding to the HEK293 cell surface, live cells were imaged a day after seeding. The cell medium was removed and 200 μL of GV with GvpC^eGFP^:RGD (OD_500_ = 2) in PBS was added for 15 min at 37 °C, then the PBS with GVs was replaced with fresh DMEM. The cells were imaged at 37 °C. Microscopic images were obtained using a Leica TCS SP5 inverted laser scanning microscope on a Leica DMI 6000 CS module equipped with an HCX Plane-Apochromat lambda blue 63× objective and a numerical aperture of 1.4 (Leica Microsystems). A 50 mW 405 nm diode laser was used for Hoechst dye (nuclear staining) excitation (emission between 420 and 460 nm), a 488 nm laser was used for eGFP excitation (emission between 502 and 563 nm), and a 10 mW 633 nm laser was used for Cholera toxin subunit B (membrane staining) excitation (emission between 648 and 721 nm).

Leica LAS AF software was used for acquisition, and ImageJ software (National Institute of Mental Health, Bethesda) was used for image processing.

### Calcium Imaging

For Ca^2+^ influx experiments, HEK293 cells with or without added GVs were incubated Fura2-TH (Setareh Biotech) and SYTO DeepRed (nuclear staining) for 1 h at 37 °C, and then the medium was replaced with fresh DMEM. Microscopic images were obtained using a Leica TCS SP5 inverted laser scanning microscope on a Leica DMI 6000 CS module equipped with an HCX Plane-Apochromat lambda blue 40× objective and a numerical aperture of 1.5 (Leica Microsystems). A 50 mW 405 nm diode laser was used for Fura2-TH (Ca^2+^ staining) excitation (emission between 406 and 460 nm), a 488 nm laser was used for eGFP excitation (emission between 502 and 563 nm), and a 10 mW 633 nm laser was used for Syto DeepRed (nuclear staining) excitation (emission between 668 and 731 nm). In the time-lapse experiments, one image was taken every 8 s. Stimulation was done with a 1 MHz ultrasound transducer (Precision Acoustics) in media from above. Images were segmented based on nuclear stain and 100 ROIs are shown in plots. Fluorescence values are normalized to the 1st minute of measurement and inverted due to Fura2-TH characteristics.

Leica LAS AF software was used for acquisition, and ImageJ software (National Institute of Mental Health, Bethesda) was used for image processing.

### Electron Microscopy

Bacterial cells were fixed using 4% formaldehyde (FA) and 2% glutaraldehyde (GA) in 0.1 M cacodylate buffer and post-fixed with 1% OsO_4_ in 0.2 M cacodylate buffer. Samples were dehydrated using increasing alcohol concentrations and embedded into Epon resin; 60 nm ultrathin sections were counterstained with U-acetate and Pb-acetate. Micrographs were taken using a TEM Philips CM100 running at 80 kV.

### Cryo-Transmission Electron Microscopy (cryo-EM)

For visualization of GVs, 3 μL of each sample was transferred to glow-discharged (GloQube Plus, Quorum, U.K.) Quantifoil 200-mesh R2/2 holey carbon grids (Quantifoil, Germany). The blot force 3 and blot time 6 s were used on a Mark IV Vitrobot (Thermo Fisher Scientific) at 4 °C and 95% humidity. Micrographs were acquired by cryo-transmission electron microscope Glacios (Thermo Fisher Scientific) operated at 200 kV and equipped with Falcon 3 direct electron detector (Thermo Fisher Scientific), with a defocus of −3 μm and at a nominal magnification of 73 000× corresponding to a pixel size of 2 Å or at a nominal magnification 6700× (Figure S2).

### Ultrasound Stimulation

24 h after transfection with the designed transcription factor and reporter plasmids (Table S2), we removed the media and added 200 μL of either GVs or PBS to the cells. After 15 min of incubation at 37 °C, 4 mL of DMEM 10% FBSs was added to the cell culture. Transfected HEK293 cells were placed on top of a 1 MHz transducer (Precision Acoustics) using a coupling gel. For the ultrasound stimulation, we used 1 MPa amplitude, 100 bursts repeated 10,000 times during 10 s. We repeated these pulses with 2 min pauses for 2 h.

### Dual Luciferase Assays

Cells were lysed 4 h after ultrasound stimulation using 200 μL of 1× Passive lysis buffer (Promega) per Petri dish, and the lysate was transferred to a 96 well. Firefly luciferase (fLuc) and Renilla luciferase (rLuc) activities were measured by using the dual luciferase assay (Promega) on a Centro microplate reader (Berthold Technologies). Relative luciferase units were calculated by normalizing fLuc to constitutive rLuc in each sample.

### Statistical Analysis

The data are presented as mean values ± sd of four independent biological repeats within the same experiment. Graphs and statistical analyses were prepared in GraphPad Prism 8. For the analysis of the GV diameters, a nested Student *t* test was used. For the analysis of ultrasound stimulation, we used the Student *t* test.

## Supporting Information Available

Buoyancy test of bacteria transformed with the plasmid with GV operon with indicated knockout genes (Figure S1); electron microscopy images of bacteria expressing GVs from plasmids with knocked-out genes (Figure S2); minimization of the GV cluster results in narrower GV width (Figure S3); effects of dilution and NaCl on the size of GV clusters (Figure S4); flow cytometry gating strategy for isolated GVs with N-tagged mCitrine (Figure S5); flow cytometry gating strategy for isolated GVs with C-terminal eGFP (Figure S6); Western blot analysis of tagged Gvp proteins (Figure S7); cryo-electron images of GvpJ^eGFP-ferritin^ GVs (Figure S8); flow cytometry gating strategy for isolated GVs and HEK293 cells with bound GVs (Figure S9); segmentation of Ca^2+^ imaging time lapse (Figure S10); summary of GV diameter measurements from cryo-electron micrographs (Table S1); summary of an impact of individual Gvp proteins coded by GV *B. megaterium* operon on formation of GVs in *E. coli* (Table S2); statistical analysis data (Table S3); list of primers used for Gvp knockout experiments (Table S4); amino acid sequences of Gvps with knockout mutations (Table S5); amounts of transfected plasmids for HEK293 cells for each 35 mm Petri dish for ultrasound stimulation (Table S6); and amino acid sequences of constructs used (Table S7) (PDF)

## Abbreviations

Cryo-EM: cryo-electron microscopy
DLS: dynamic light scattering
EM: electron microscopy
GV: gas vesicle
Gvp: gas vesicle protein
HEK293: human embryonic kidney
RLU: relative light units
US: ultrasound
