# Supplementary material for: Protein Gas Vesicles of Bacillus megaterium as Enhancers of Ultrasound-Induced Transcriptional Regulation
Source: ACS Nano. 2024 Jun 19;18(26):16692–700. doi: 10.1021/acsnano.4c01498 (PMC11223475; doi:10.1021/acsnano.4c01498)
Supplement: Supplementary file 1 — nn4c01498_si_001.pdf [file nn4c01498_si_001.pdf]

Protein gas vesicles of *Bacillus megaterium* as enhancers of ultrasound-induced transcriptional regulation

Supporting information

Vid Jazbec*[1], Nina Varda[1], Ernest Šprager[1], Maja Meško[1], Sara Vidmar[1], Rok Romih[2], Marjetka Podobnik[3], Andreja Kežar[3], Roman Jerala[1,4], Mojca Benčina[1,4,5+]

[1] Department of Synthetic Biology and Immunology, National Institute of Chemistry, 1000 Ljubljana, Slovenia
[2] Institute of Cell Biology, Faculty of Medicine, University of Ljubljana, 1000 Ljubljana, Slovenia
[3] Department of Molecular Biology and Nanobiotechnology, National Institute of Chemistry, 1000 Ljubljana, Slovenia
[4] CTGCT, Centre of Technology of Gene and Cell Therapy, Hajdrihova 19, 1000 Ljubljana, Slovenia
[5] University of Ljubljana, Kongresni trg 12, 1000 Ljubljana, Slovenia

* First author
+ Corresponding author

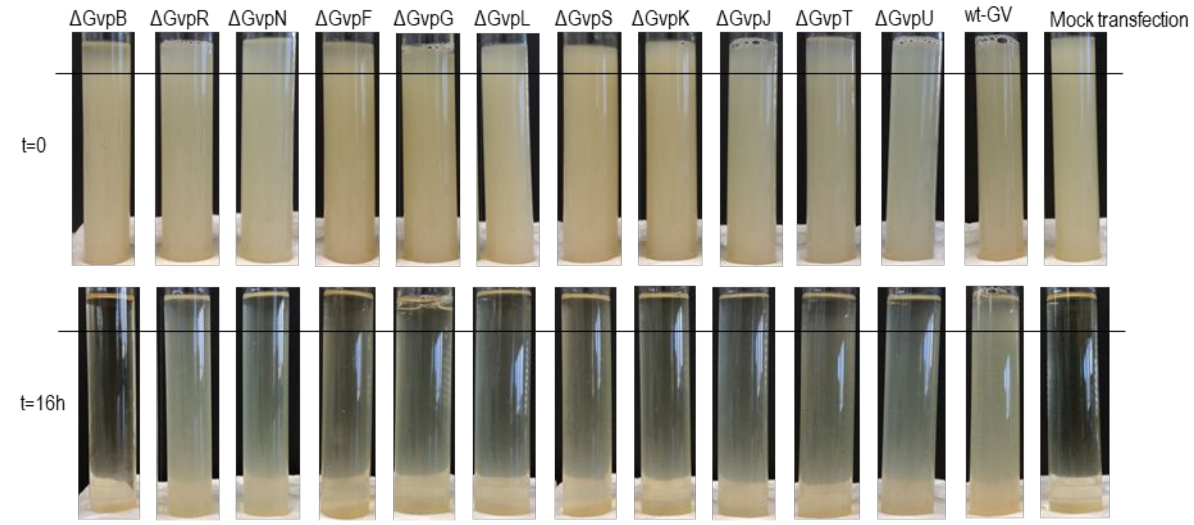

Figure S1. Buoyancy test of bacteria transformed with the plasmid with GV operon with indicated knock-out genes. Images at time 0 (above) and 16 h later (below) are shown. The line represents the depth at which samples for OD600 measurements were taken.

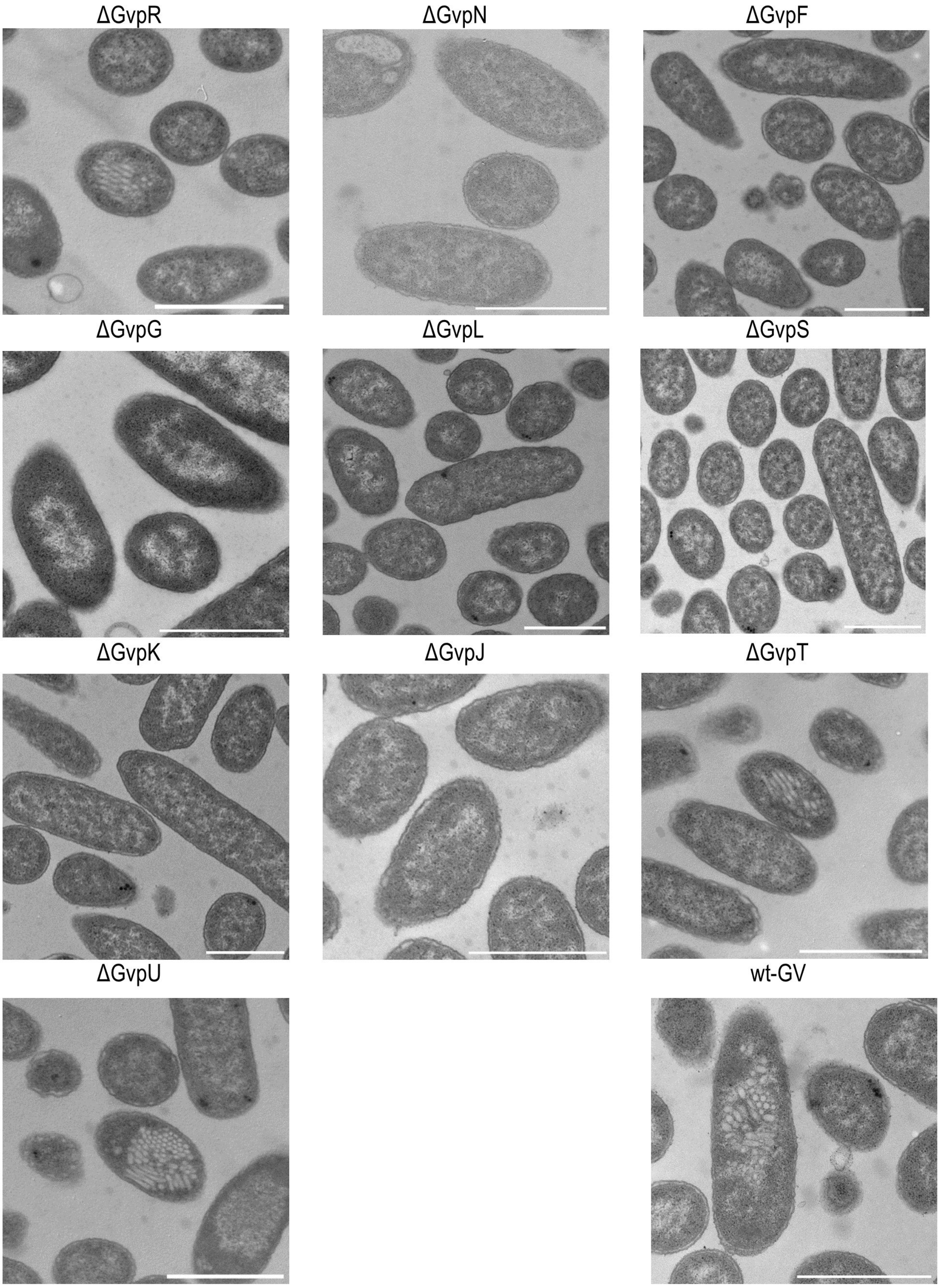

Figure S2. Electron microscopy images of bacteria expressing GVs from plasmids with knocked out genes. Note: Gas vesicles (GVs) were observed exclusively in bacteria expressing GVs from the ΔGvpR, T or U cluster, and wt-GV. Conversely, bacteria transformed with the ΔGvpF, G, L, S, K or J cluster exhibited no GV formation. Although GV formation from the ΔGvpN cluster was not detected, flotation assays indicated that GvpN is not essential for flotation. The white scale bars represent 1μm

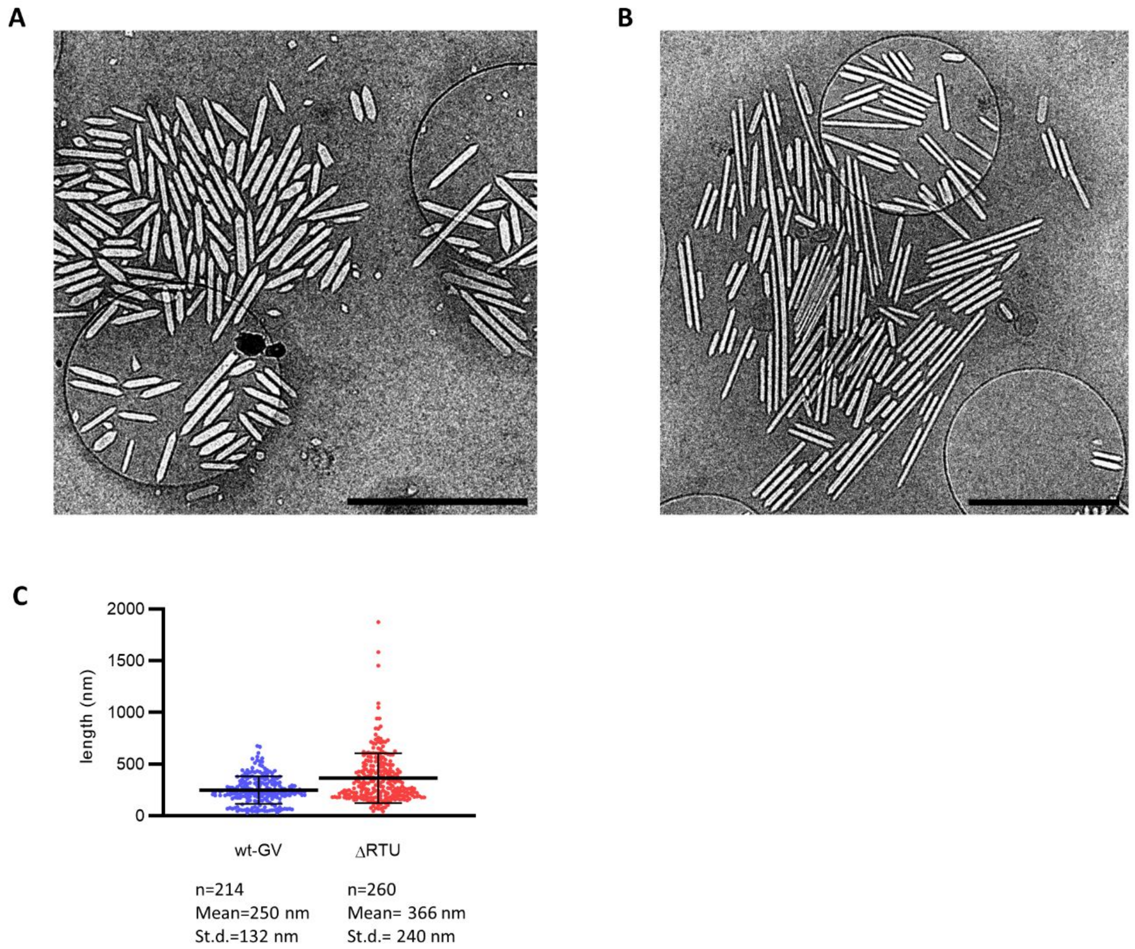

Figure S3. Minimization of the GV cluster results in narrower GV width. The GVs isolated from *E. coli* transformed with the plasmid encoding full wt-GV operon (**A**) or a plasmid with a minimized ΔRTU cluster (**B**) were purified and imaged with cryo-EM. The black bar represents 1 µm. **(C)** The diameters of GVs with mean ± s.d. (n = number of measured vesicles combined from multiple cryo-EM experiments).

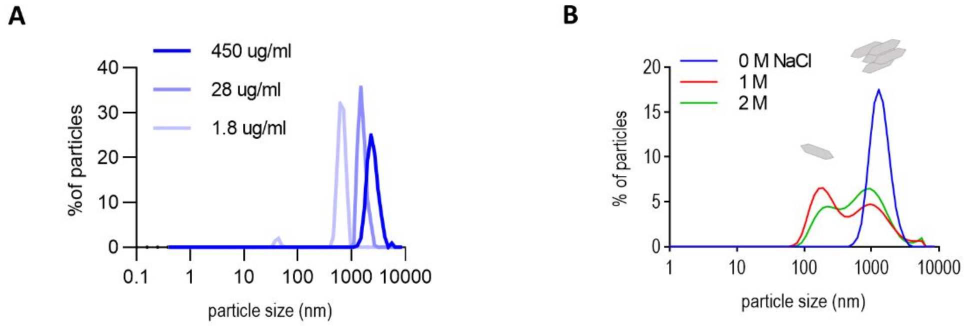

Figure S4. The effects of dilution and NaCl on the size of wt-GV clusters. A The effect of dilution on the size of GV clusters. The GVs were diluted with PBS. **B** The effect of NaCl on the size of GV clusters. The concentration of GVs was 10 µg/ml. The curves in show the average value of three measurements of wt-GVs using DLS.

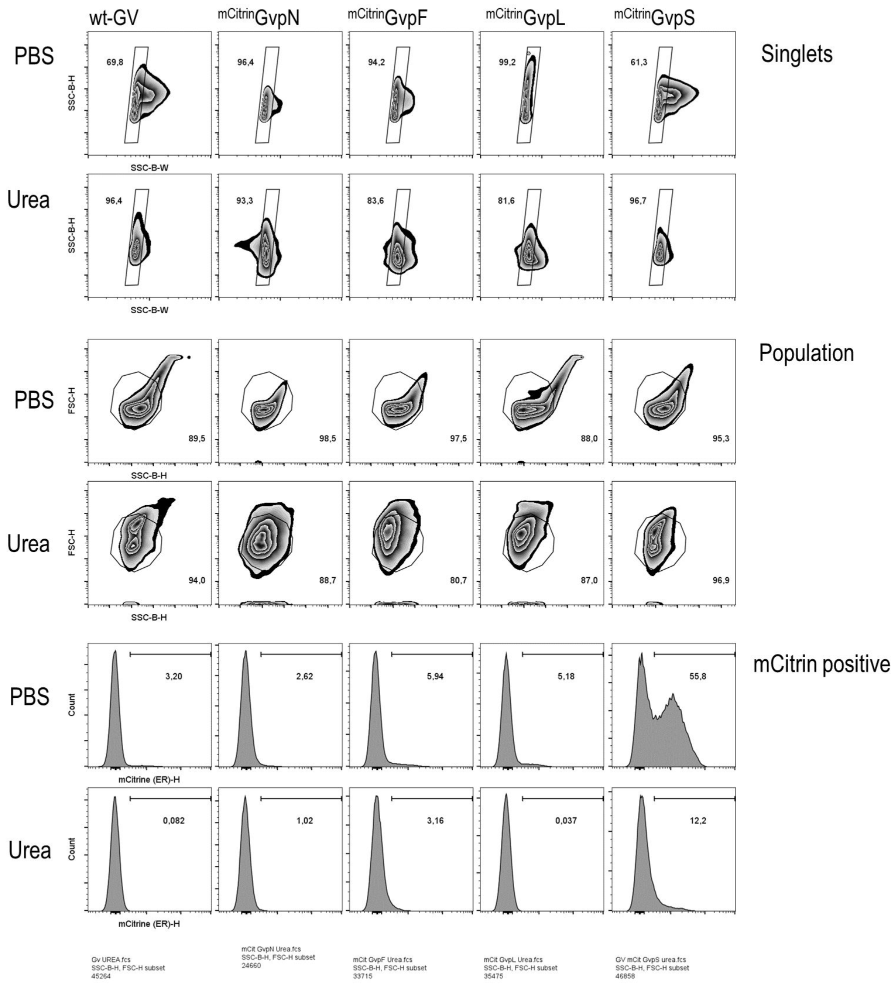

Figure S5. Flow cytometry gating strategy for isolated GVs with N-tagged mCitrine.

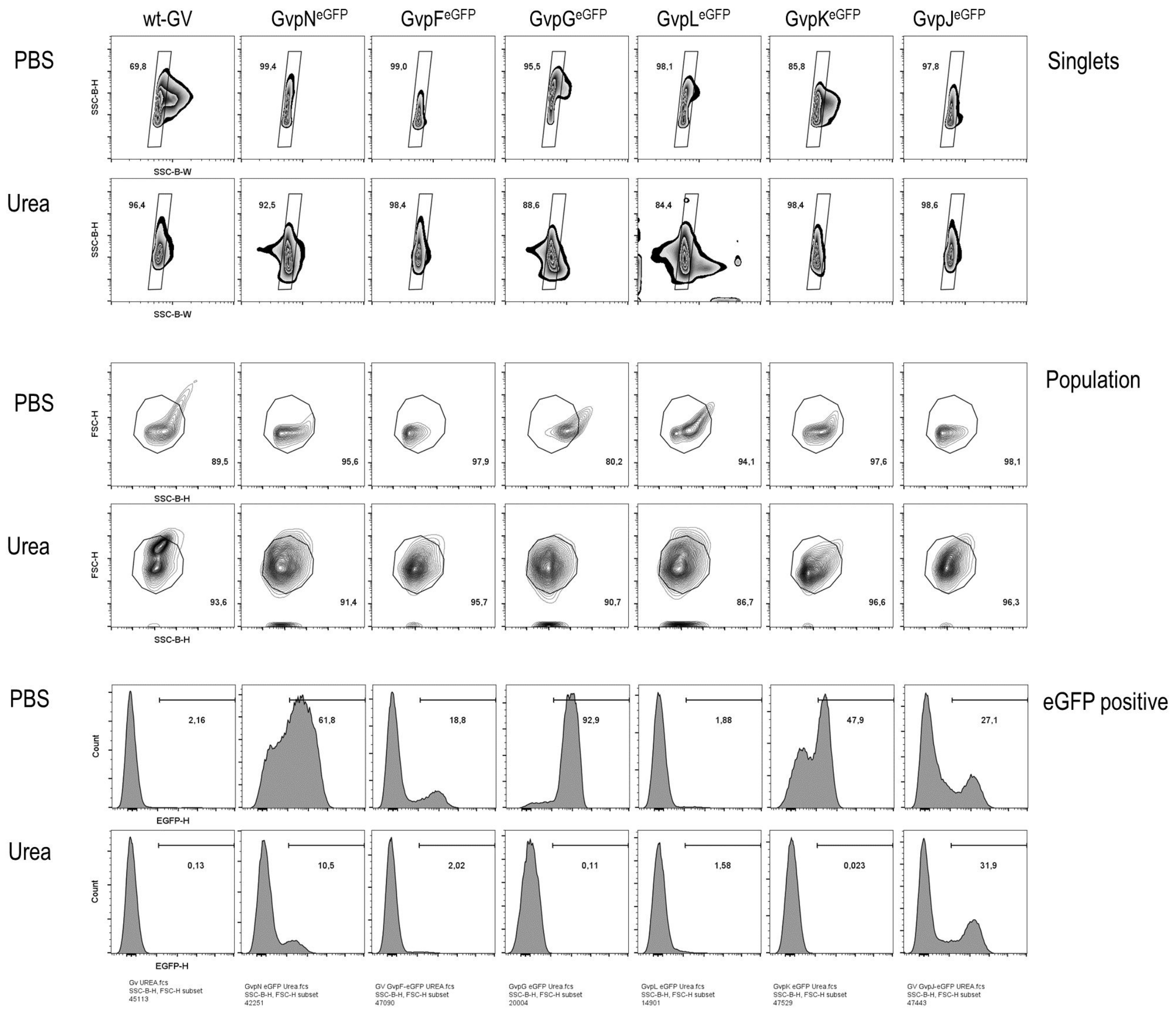

Figure S6. Flow cytometry gating strategy for isolated GVs with C-terminal eGFP.

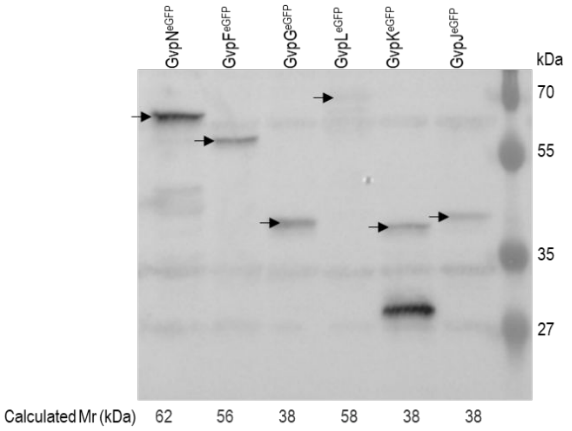

Figure S7. Western blot analysis of tagged Gvp proteins detected with antiGFP antibodies. The GVs were denatured with 10 min incubation at 95 °C with a reducing loading buffer.

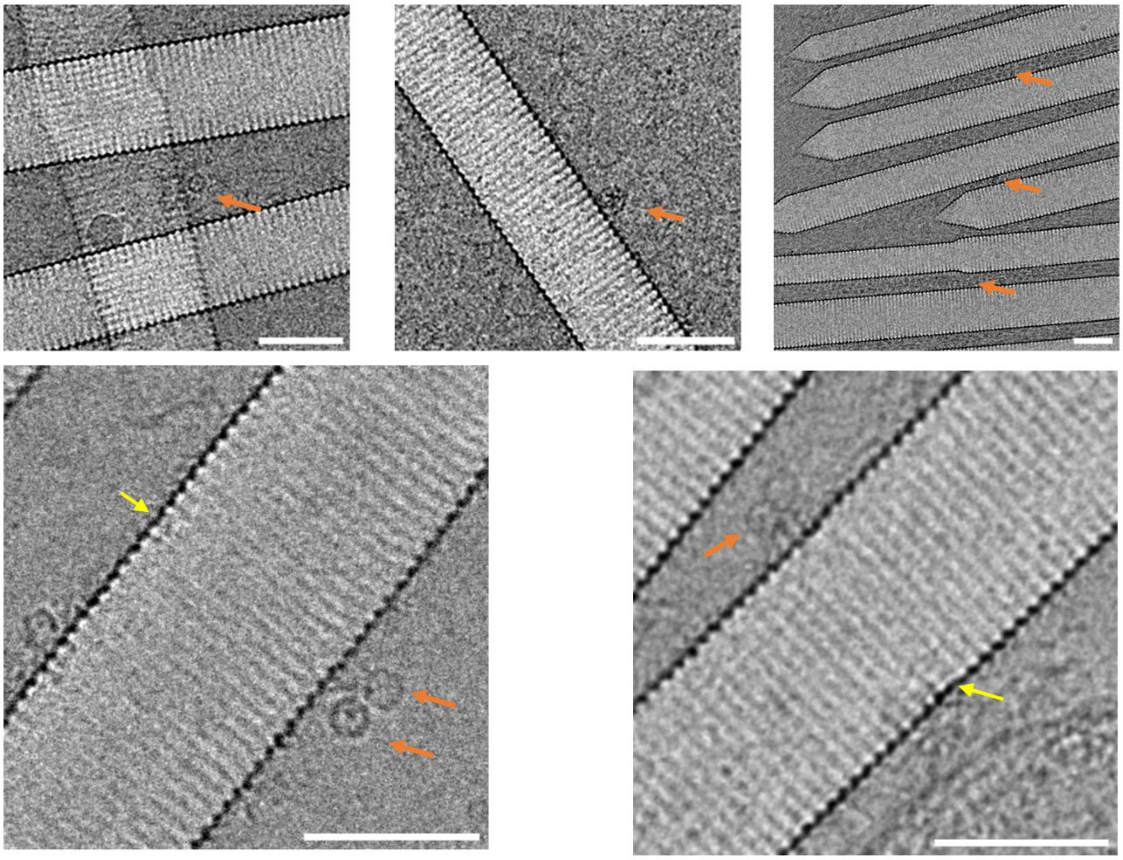

Figure S8. **Cryo-electron images of GvpJ$^{eGFP\text{-}ferritin}$ GVs.** GVs were isolated from bacterial cells and purified using 1 M urea before they were observed under cryo-EM. The orange arrows point to apoferritin structures. The yellow arrows on the bottom images show the polarity inversal point where the two half-shells of the GVs link. The white scale bar represents 50 nm.

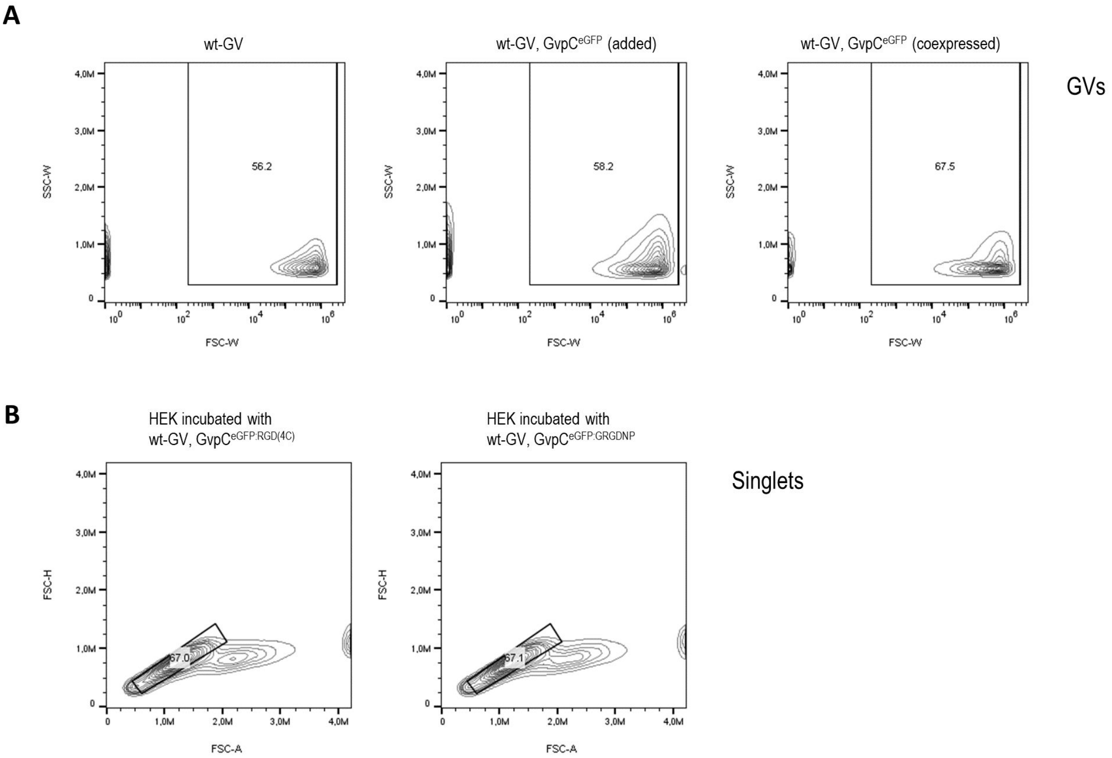

Figure S9. **Flow cytometry gating strategy for isolated GVs and HEK293 cells with bound GVs**. (A) Gating strategy for isolated wt-GVs, wt-GV with coexpressed GvpC^{eGFP} or wt-GVs with added GvpC^{eGFP}. (B) Gating strategy of HEK293 cells incubated with GVs with GvpC$^{eGFP:RGD(4C)}$ or GvpC$^{eGFP:GRGDNP}$.

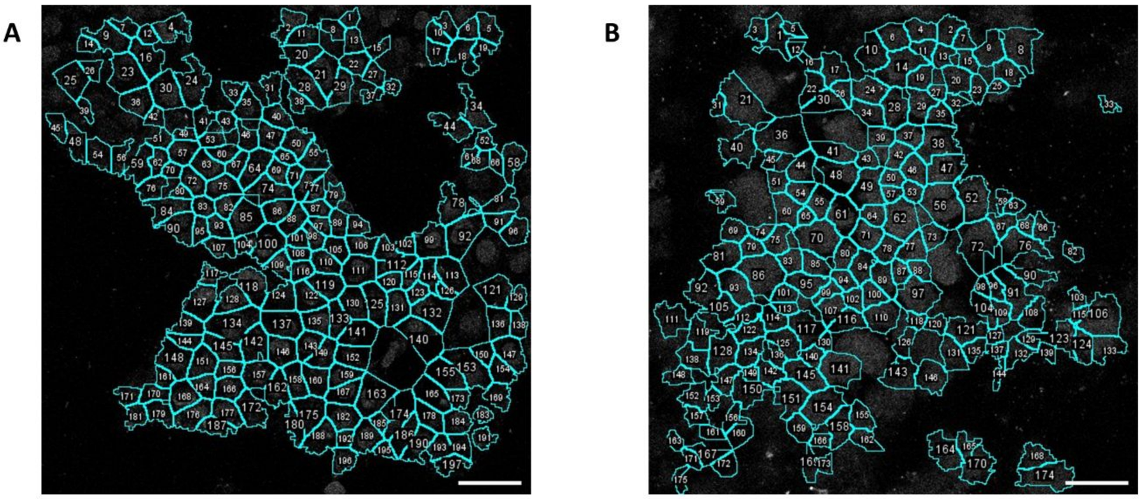

Figure S10. Segmentation of Ca^{2+} imaging time-lapse. Overlay of ROIs based on a nuclear stain on Fura2-TH channel for HEK293 (**A**) and HEK293 cells with added GVs (**B**). Scale bar represent 20 µm.

Supplementary Tables

Table S1.Summary of GV diameter measurements from cryo-electron micrographs.

	GV with									
	wt-GV	GvpN^{eGFP}	GvpF^{eGFP}	GvpG^{eGFP}	GvpL^{eGFP}	GvpK^{eGFP}	GvpJ^{eGFP}	^{mCitrin}GvpF	^{mCitrin}GvpL	^{mCitrin}GvpS
Number of GV	330	97	88	163	111	102	74	155	102	123
Mean (nm)	48,8	49,6	50,5	50,5	52,5	47,9	50,4	51,7	52,5	50,6

Table S2. Summary of an impact of individual Gvp proteins coded by GV *B. megaterium* operon on formation of GVs in *E. coli*.

Gvp	Floating assay	$\mathrm{Gvp}^{\mathrm{eGFP}}$				$^{\mathrm{mCitrin}}\mathrm{Gvp}$			
		Expression	Binding		Cryo-EM	Expression	Binding		Cryo-EM
			PBS	6M urea			PBS	6M urea	
B	-	np				np			
R	+	np				np			
N	+	+	+	+/-	as wt-GV		-	-	bicones
F	-	+	+	-	as wt-GV		-		wider
G	-	+	+	-	as wt-GV	*			
L	-	+/-	-	-	wider		-	-	wider
S	-	np					+	-	as wt-GV
K	-	+	+	-	as wt-GV	np			
J	-	+	+	+	as wt-GV	*			
T	+	np				np			
U	+	np				np			

np, not prepared; * no GV obtained

Table S3. Statistical analysis data.

Figures	Test details	Significance	Summary	P value
**2 C**	Nested t test			
	wt-GV vs ΔRTU	Yes	*	0,0214
**2 D**	Paired t test	Significance		P value
	wt-GV vs ΔRTU 450 ug/ml	No		0.357071
	wt-GV vs ΔRTU 225 ug/ml	No		0.305480
	wt-GV vs ΔRTU 112.5 ug/ml	No		0.301717
	wt-GV vs ΔRTU 56.25 ug/ml	No		0.049412
	wt-GV vs ΔRTU 28.125 ug/ml	Yes		0.005480
	wt-GV vs ΔRTU 14.0625 ug/ml	No		0.058419
	wt-GV vs ΔRTU 7.03125 ug/ml	No		0.093146
	wt-GV vs ΔRTU 3.515625 ug/ml	No		0.752680
	wt-GV vs ΔRTU 1.757813 ug/ml	Yes		0.002572
	wt-GV vs ΔRTU 0.878906 ug/ml	Yes		0.000336
	wt-GV vs ΔRTU 0.439453 ug/ml	No		0.040227
	wt-GV vs ΔRTU 0.219727 ug/ml	Yes		0.000019
**3 B, 3 D**	One-way ANOVA multiple comparisons test			
	wt-GV vs. GvpN-eGFP	No	ns	0,9686
	wt-GV vs. GvpF-eGFP	No	ns	0,3701
	wt-GV vs. GvpG-eGFP	No	ns	0,1142
	wt-GV vs. GvpL-eGFP	Yes	****	<0,0001
	wt-GV vs. GvpK-eGFP	No	ns	0,9189
	wt-GV vs. GvpJ-eGFP	No	ns	0,5351
	wt-GV vs. mCitrine-GvpF	Yes	***	0,0003
	wt-GV vs. mCit-GvpL	Yes	****	<0,0001
	wt-GV vs. mCit-GvpS	No	ns	0,1448
**4 C**	Nested t test			
	wt-GV vs GV+C	No	ns	0,0674
**5 D**	Unpaired t-test, two-tailed			
	Control vs US	Yes	*	0,0265
	GV vs GV and US	Yes	*	0,0114
	US vs GV and US	Yes	*	0,0147

Table S4. List of primers used for Gvp knock-out experiments.

PRIMER NAME	OLIGONUCEOTIDE SEQUENCE
**DEL_GVPB-F**	gagatatgtctattTaaaaaagtactaatag
**DEL_GVPB-R**	ctattagtacttttttAaatagacatatctc
**DEL_GVPR-F**	ctaaaaatggaaattTaaaaaattatgcaag
**DEL_GVPR-R**	cttgcataatttttttAaatttccatttttag
**DEL_GVPN-F**	gtaaaaatgaccgtctAaacagacaaaagg
**DEL_GVPN-R**	ccttttgtctgttTagacggtcatttttac
**DEL_GVPF-F**	gaaaacatgagtgaaTAaaacgaaacagg
**DEL_GVPF-R**	cctgtttcgttttTAttcactcatgttttc
**DEL_GVPG-F**	gaaataacgtgcttcacTaattagtaaccgcac
**DEL_GVPG-R**	gtgcggttactaattAgtgaagcacgttatttc
**DEL_GVPL-F**	cctagatgggagaatAactgtatttatacg
**DEL_GVPL-R**	cgtataaatacagtTattctcccatctagg
**DEL_GVPS-F**	cattatgtctcttTaacaatccatggag
**DEL_GVPS-R**	ctccatggattgttAaagagacataatg
**DEL_GVPK-F**	ggatgcaaccggtcagcTaagcaaatggacg
**DEL_GVPK-R**	cgtccatttgcttAgctgaccggttgcatcc
**DEL_GVPJ-F**	gaacagtatggcagtcTaacataatatgcag
**DEL_GVPJ-R**	ctgcatattatgttAgactgccatactgttc
**DEL_GVPT-F**	gatttacaatggcaactTaaacaaaattagataac
**DEL_GVPT-R**	gttatctaattttgtttAagttgccattgtaaatc
**DEL_GVPU-F**	gaaagacatgagtacaTAAggcccttctttttc
**DEL_GVPU-R**	gaaaaagaagggccTTAtgtactcatgtctttc

Table S5. Aminoacid sequences of Gvps with knockout mutations

Amino acids which codons were mutated to STOP codons are highlighted in red. In case of GvpU a stop codon was inserted at location designated with *.

Protein	Oligonuceotide sequence
**GvpB**	MSIQKSTNSSSLAEVIDRILDKGIVIDAFARVSVVGIEILTIEARVVIASVDTWLRYAEAVGLLRD<br>DVEENGLPERSNSSEGQPRFSI*
**GvpR**	MEIKKIMQAVNDFFGEHVAPPHKITSVEATEDEGWRVIVEVIEEREYMKKYAKDEMLGTYECF<br>VNKEKEVISFKRLDVRYRSAIGIEA*
**GvpN**	MTVLTDKRKKGSGAFIQDDETKEVLSRALSYLKSGYSIHFTGPAGGGKTSLARALAKKRKRPV<br>MLMHGNHELNNKDLIGDFTGYTSKKVIDQYVRSVYKKDEQVSENWQDGRLLEAVKNGYTLIY<br>DEFTRSKPATNNIFLSILEEGVLPLYGVKMTDPFVRVHPDFRVIFTSNPAEYAGVYDTQDALLDR<br>LITMFIDYKDIDRETAILTEKTDVEEDEARTIVTLVANVRNRSGDENSSGLSLRASLMIATLATQQ<br>DIPIDGSDEDFQTLCIDILHHPLTKCLDEENAKSKAEKIILEECKNIDTEEK*
**GvpF**	MSETNETGIYIFSAIQTDKDEEFGAVEVEGTKAETFLIRYKDAAMVAAEVPMKIYHPNRQNLLM<br>HQNAVAAIMDKNDTVIPISFGNVFKSKEDVKVLLENLYPQFEKLFPAIKGKIEVGLKVIGKKEW<br>LEKKVNENPELEKVSASVKGKSEAAGYYERIQLGGMAQKMFTSLQKEVKTDVFSPLEEAAEAA<br>KANEPTGETMLLNASFLINREDEAKFDEKVNEAHENWKDKADFHYSGPWPAYNFVNIRLKVEE<br>K*
**GvpG**	VLHKLVTAPINLVVKIGEKVQEEADKQLYDLPTIQQKLIQLQMMFELGEIPEEAFQEKEDELLM<br>RYEIAKRREIEQWEELTQKRNEES*
**GvpL**	MGELLYLYGLIPTKEAAAIEPFPSYKGFDGEHSLYPIAFDQVTAVVSKLDADTYSEKVIQEKME<br>QDMSWLQEKAFHHHETVAALYEEFTIIPLKFCTIYKGEESLQAAIEINKEKIENSLTLLQGNEEW<br>NVKIYCDDTELKKGISETNESVKAKKQEISHLSPGRQFFEKKKIDQLIEKELELHKNKVCEEIHDK<br>LKELSLYDSVKKNWSKDVTGAAEQMAWNSVFLLPSLQITKFVNEIEELQQRLENKGWKFEVTG<br>PWPPYHFSSFA*
**GvpS**	MSLKQSMENKDIALIDILDVILDKGVAIKGDLIISIAGVDLVYLDLRVLISSVETLVQAKEGNHKP<br>ITSEQFDKQKEELMDATGQPSKWTNPLGS*
**GvpK**	MQPVSQANGRIHLDPDQAEQGLAQLVMTVIELLRQIVERHAMRRVEGGTLTDEQIENLGIALM<br>NLEEKMDELKEVFGLDAEDLNIDLGPLGSLL*
**GvpJ**	MAVEHNMQSSTIVDVLEKILDKGVVIAGDITVGIADVELLTIKIRLIVASVDKAKEIGMDWWEN<br>DPYLSSKGANNKALEEENKMLHERLKTLEEKIETKR*
**GvpT**	MATETKLDNTQAENKENKNAENGSKEKNGSKASKTTSSGPIKRAVAGGIIGATIGYVSTPENRK<br>SLLDRIDTDELKSKASDLGTKVKEKSKSSVASLKTSAGSLFKKDKDKSKDDEENVNSSSSETED<br>DNVQEYDELKEENQTLQDRLSQLEEKMNMLVELSLNKNQDEEAEDTDSDEEENDENDENDEN<br>EQDDENEEETSKPRKKDKKEAEEEESESDEDSEEEEEDSRSNKKNKKVKTEEEDEDESEEEKKE<br>AKPKKSTAKKSKNTKAKKNTDEEDDEATSLSSEDDTTA*
**GvpU**	MST*GPSFSTKDNTLEYFVKASNKHGFSLDISLNVNGAVISGTMISAKEYFDYLSETFEEGSEVA<br>QALSEQFSLASEASESNGEAEAHFIHLKNTKIYCGDSKSTPSKGKIFWRGKIAEVDGFFLGKISDA<br>KSTSKKSS*

Table S6. Amounts of transfected plasmids for HEK293 cells for each 35 mm petri dish for ultrasound stimulation. Empty pcDNA3 backbone was used to equalize amount of transfected plasmids to 1800 ng.

**Input plasmid**	**Amount (ng)**
Figure 5B	
mNFAT-TALA-VP16-KRΦ	10
$_{10TALE}$–Pmin-fLuc	1000
phRL-TK	30

Table S7. Amino acid sequences of constructs used.

**Aminoacid sequence**
**^{mCitrine}Gvp (the provided sequence was C terminally tagged to Gvps in the plasmid pST39-pNL29)**
**>mCitrine**<br>MVSKGEELFTGVVPILVELDGDVNGHKFSVSGEGEGDATYGKLTLKFICTTGKLPVPWPTLVTTFGYGLMCFARYPDHMKQHDFFKSAMP<br>EGYVQERTIFFKDDGNYKTRAEVKFEGDTLVNCIELKGIDFKEDGNILGHKLEYNYNSHNVYIMADKQKNGIKVNFKIRHNIEDGSVQLADH<br>YQQNTPIGDGPVLLPDNHYLSYQSALSKDPNEKRDHMVLLEFVTAAGITLGMDELYK<br>**>GS4 linker**<br>SGSG
**Gvp^{eGFP} (the provided sequence was C terminally tagged to Gvps in the plasmid pST39-pNL29)**
**>GS4 linker**<br>SGSG<br>**>eGFP**<br>VSKGEELFTGVVPILVELDGDVNGHKFSVSGEGEGDATYGKLTLKFICTTGKLPVPWPTLVTTLTYGVQCFSRYPDHMKQHDFFKSAMPE<br>GYVQERTIFFKDDGNYKTRAEVKFEGDTLVNRIELKGIDFKEDGNILGHKLEYNYNSHNVYIMADKQKNGIKVNFKIRHNIEDGSVQLADHY<br>QQNTPIGDGPVLLPDNHYLSTQSALSKDPNEKRDHMVLLEFVTAAGITLGMDELYK
**GvpJ$^{eGFP-ferritin}$ (the provided sequence was C terminally tagged to GvpJ in the plasmid pST39-pNL29)**
**>GS4 linker**<br>SGSG<br>**>eGFP**<br>VSKGEELFTGVVPILVELDGDVNGHKFSVSGEGEGDATYGKLTLKFICTTGKLPVPWPTLVTTLTYGVQCFSRYPDHMKQHDFFKSAMPE<br>GYVQERTIFFKDDGNYKTRAEVKFEGDTLVNRIELKGIDFKEDGNILGHKLEYNYNSHNVYIMADKQKNGIKVNFKIRHNIEDGSVQLADHY<br>QQNTPIGDGPVLLPDNHYLSTQSALSKDPNEKRDHMVLLEFVTAAGITLGMDELYK<br>**>GSGLE linker**<br>GSGLE<br>**>ferritin**<br>ESQVRQQFSKDIEKLLNEQVNKEMQSSNLYMSMSSWCYTHSLDGAGLFLFDHAAEEYEHAKKLIIFLNENNVPVQLTSISAPEHKFEGLTQ<br>IFQKAYEHEQHISESINNIVDHAIKSKDHATFNFLQWYVAEQHEEEVLFKDILDKIELIGNENHGLYLADQYVKGIAKSRKS
**GvpC$^{eGFP\ RGD(4C)}$**
**>his tag**<br>MHHHHHH<br>**>GvpC**<br>GISLMAKIRQEHQSIAEKVAELSLETREFLSVTTAKRQEQAEKQAQELQAFYKDLQETSQQFLSETAQARIAQAEKQAQELLAFHKELQETS<br>QQFLSATAQARIAQAEKQAQELLAFYQEVRETSQQFLSATAQARIAQAEKQAQELLAFHKELQETSQQFLSATADARTAQAKEQKESLLKF<br>RQDLFVSIFG<br>**>GSGLE linker**<br>GSGLE<br>**>eGFP**<br>VSKGEELFTGVVPILVELDGDVNGHKFSVSGEGEGDATYGKLTLKFICTTGKLPVPWPTLVTTLTYGVQCFSRYPDHMKQHDFFKSAMPE<br>GYVQERTIFFKDDGNYKTRAEVKFEGDTLVNRIELKGIDFKEDGNILGHKLEYNYNSHNVYIMADKQKNGIKVNFKIRHNIEDGSVQLADHY<br>QQNTPIGDGPVLLPDNHYLSTQSALSKDPNEKRDHMVLLEFVTAAGITLGMDELYK<br>**>GSGLE linker**<br>GSGLE<br>**>RGD (4C)**<br>CDCRGDCFC
**GvpC$^{eGFP\ GRGDNP}$**
**>his tag**<br>MHHHHHH<br>**>GvpC**<br>GISLMAKIRQEHQSIAEKVAELSLETREFLSVTTAKRQEQAEKQAQELQAFYKDLQETSQQFLSETAQARIAQAEKQAQELLAFHKELQETS<br>QQFLSATAQARIAQAEKQAQELLAFYQEVRETSQQFLSATAQARIAQAEKQAQELLAFHKELQETSQQFLSATADARTAQAKEQKESLLKF<br>RQDLFVSIFG<br>**>GSGLE linker**<br>GSGLE<br>**>eGFP**<br>VSKGEELFTGVVPILVELDGDVNGHKFSVSGEGEGDATYGKLTLKFICTTGKLPVPWPTLVTTLTYGVQCFSRYPDHMKQHDFFKSAMPE<br>GYVQERTIFFKDDGNYKTRAEVKFEGDTLVNRIELKGIDFKEDGNILGHKLEYNYNSHNVYIMADKQKNGIKVNFKIRHNIEDGSVQLADHY<br>QQNTPIGDGPVLLPDNHYLSTQSALSKDPNEKRDHMVLLEFVTAAGITLGMDELYK<br>**>GSGLE linker**

GSGLE
>GRGDNP
GRGDNP
